# Two fetuses in one family of arterial tortuosity syndrome: prenatal ultrasound diagnosis

**DOI:** 10.1186/s12884-021-03960-w

**Published:** 2021-08-12

**Authors:** Meiling Liang, Huaxuan Wen, Shengli Li

**Affiliations:** Department of Ultrasound, Affiliated Shenzhen Maternity & Child Healthcare Hospital, Southern Medical University, Shenzhen, 518028 China

**Keywords:** Arterial tortuosity syndrome, Prenatal ultrasound diagnosis, Cutis laxa, Pulmonary artery sling

## Abstract

**Background:**

Arterial tortuosity syndrome (ATS) is a rare autosomal recessive connective tissue disorder chiefly characterized by elongated and tortuosity of the large and medium sized arteries and anomalies of the vascular elastic fibers. Here we reported cases of brother about ATS from the same family on the prenatal ultrasound diagnosis. Reports of this case are rare in antenatally and we draw the vessel simulated diagram to display visually.

**Case presentation:**

Prenatal ultrasound scanning at 29 weeks of gestation of the first fetus showed obvious tortuous and elongated of the aortic arch, ductus arteriosus, left and right pulmonary arteries, carotid and subclavian arteries. Three months after delivery, Contrast-enhanced computed tomography images (CTA) were performed to clearly display vascular abnormalities consistent with prenatal diagnosis of ultrasound. Whole exome sequencing (WES) was performed eight months after birth, two heterozygous variants of SLC2A10 gene was detected in newborn and their father and mother, respectively. Prenatal ultrasound scan at 22 weeks of gestation of the second fetus showed similar cardiovascular imaging. After birth the siblings have facial characteristic features gradually as aging. No surgical intervention was performed in the siblings follow up 19 months.

**Conclusions:**

The key points of prenatal ultrasound diagnosis of ATS are the elongation and tortuosity of the large and medium sized arteries. Genetic counseling is the process of providing individuals and families with information on the nature, inheritance, and implications of genetic disorders to help them make informed medical and personal decisions.

**Supplementary Information:**

The online version contains supplementary material available at 10.1186/s12884-021-03960-w.

## Background

Arterial tortuosity syndrome (ATS) is a rare autosomal recessive connective tissue disorder chiefly characterized by elongation and tortuosity of the large and medium sized arteries and anomalies of the vascular elastic fibers, and a propensity for arterial and aortic aneurysm formation with vascular dissection, caused by mutations in SLC2A10 [1, 2]. The first few years of life seem to be the most critical for potentially life-threatening events, particularly acute respiratory insufficiency and other complications related to pulmonary artery stenosis. Here we reported two cases of brothers about ATS from the same family on the prenatal ultrasound diagnosis.

## Case presentation

**Fig. 1:**
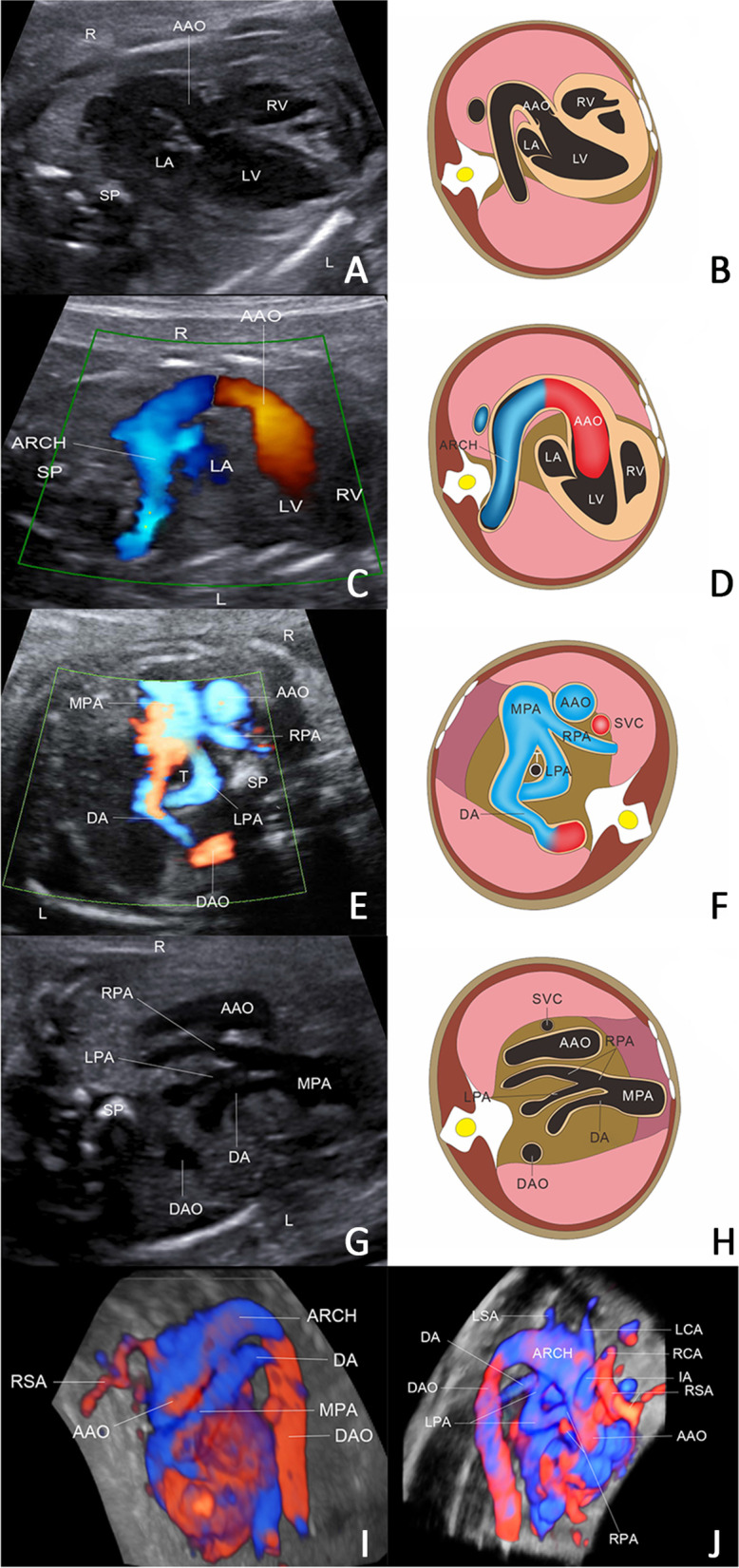
Prenatal ultrasound images and simulation of the first fetus at 29 weeks. **A**, **C** Tortuous of the AAO and aortic arch in the Left ventricular outflow tract plane and Color Doppler. **E**, **G**. DA, LPA and RPA were significantly longer and tortuous. The LPA originated from the MPA and passed in front of the trachea and esophagus in the three vessel sections and Color Doppler. **I**, **J**. The AAO, aortic arch, LPA and RSA were significantly longer and tortuous in the horizontal section of the HD flow imaging. **B**, **D**, **F**, **H**. The monograms of **A**, **C**, **E**, **G** respectively. The measurement in detail as follows: AAO 0.51 cm, Aortic Arch 0.49 cm, MPA 0.61 cm, RPA 0.26 cm, LPA0.27, DA0.26 cm. AAO: ascending aorta; RSA: right subclavian artery; RV: right ventricle; LV: left ventricle; LA: left atrium; DAO: descending aorta; DA: ductus arteriosus; MPA: main pulmonary artery; SP: spine

A 29 weeks fetus suspected fetal heart abnormality was referred to our center for the detailed prenatal ultrasound scanning. Routine scanning demonstrated that the fetus was in good status without any extra-cardiac abnormalities. Echocardiography showed (Fig. 1) obviously tortuous and elongated of the great arteries include Arch, DA, LPA, RPA, LCA, RCA, LSA and RSA. An additional movie files show this in more detail. (See Additional files 1, 2 and 3: Video) Given the echocardiographic findings suggestive of a connective tissue disorder, genetic diagnosis was recommended but the gravida refuse all the invasive examination.

The newborn presented with respiratory distress after birth. Umbilical cord blood was performed chromosome microarray detection at the time of delivery in the first neonate, indicating no abnormalities of sex chromosome and autosomes with definite clinical significance. Postpartum CT diagnosis: pulmonary hypertension, left and right ventricular wall thickening, the aorta, PA and cervical artery were tortuous, and clinically suspected Marfan syndrome.

**Fig. 2:**
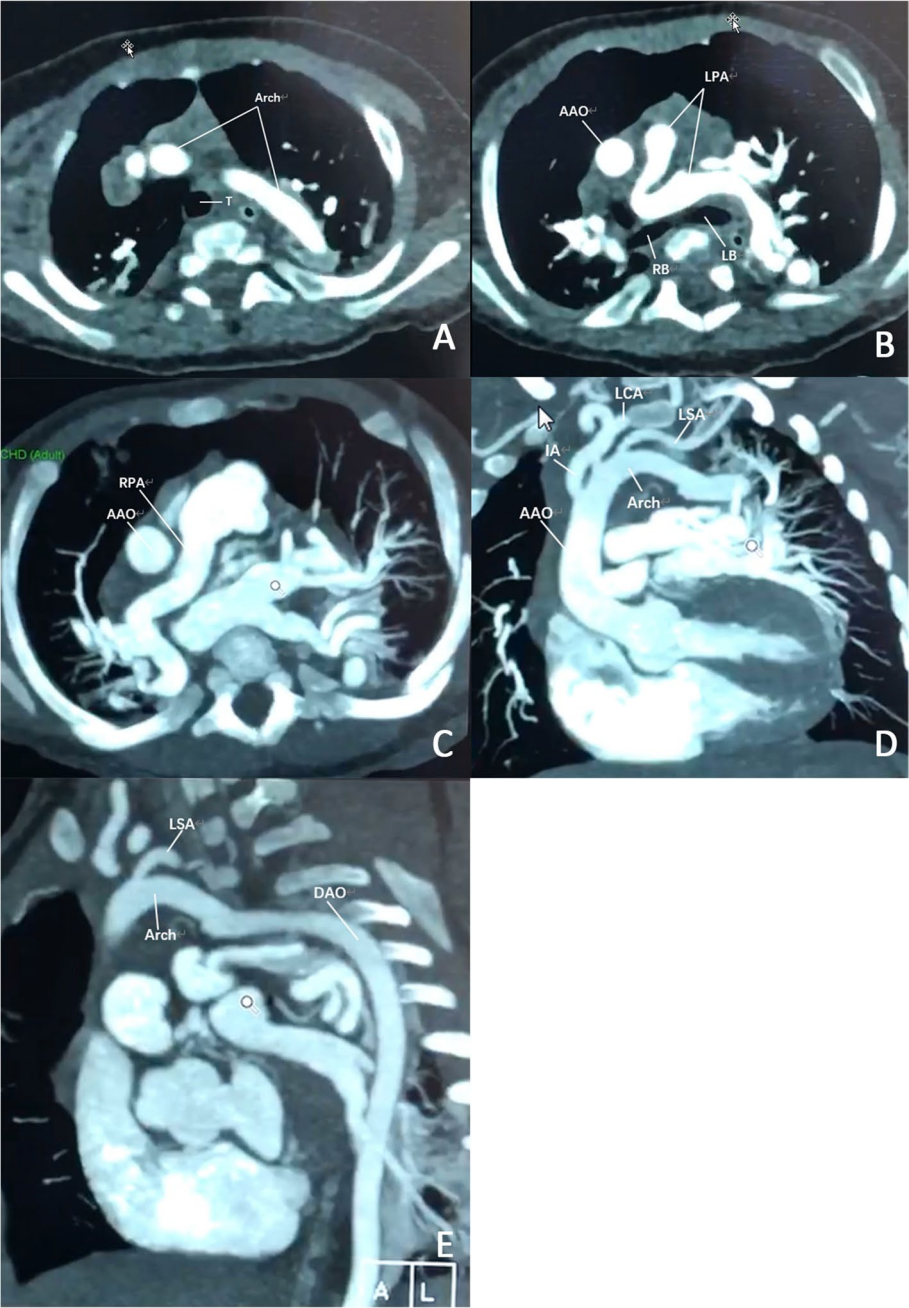
Contrast-enhanced computed tomography images of the older brother three months after delivery. **A** The CTA cross-sectional reveal the long aortic Arch in front of the trachea. **B** LPA are tortuous and dilated in front of the left and right bronchus. **C** RPA originated from MPA and also tortuous and dilated, the dot refers pulmonary vein. **D** Sagittal section of the aortic arch reveals three branches of Arch (IA, LCA, LSA) are tortuous. **E** The sagittal section of the descending aorta shows a very large vascular span of the DAO. Arch: aortic arch; AAO: ascending aorta; RPA: right pulmonary artery; LPA: left pulmonary artery; IA: innominate artery; LCA: left common carotid artery; LSA: left subclavian artery; DAO: descending aorta; T: trachea; LB: left bronchus; RB: right bronchus

Three months after delivery in the first neonate, CTA was performed to clearly display vascular abnormalities. The MPA send out RPA and LPA, LPA distinctly tortuous and elongated through the front of the trachea. The aortic arch arises from the ascending aorta and pass backward to the left of the trachea and esophagus to join the descending aorta which lies farther than normal aortic arch system of man [3]. A left aortic arch which gives rise to the IA, LCA and LSA in that order, but they are all tortuous exactly (Fig. 2). Whole exome sequencing(WES)was performed 8 months after birth in the first baby, two heterozygous variants of SLC2A10 gene c.1057_1058delCT (p. L353Tfs*8) and c.912 T > G (p.C304W) were detected (Table 1) and sequencing data showed that the two mutations were inherited from the mother and father (both heterozygous). P. L353TFS *8 is a rare code shift mutation predicts that protein synthesis may lead to premature termination of amino acids. P.c304w is a rare missense variation, and the variation region is an important part of this protein. Neither mutation has been reported in the associated clinical cases. So far, these two variants are relatively infrequent in our reference population gene database. In summary, according to the American Guidelines for classification of ACMG variants (PMID: 25,741,868), the two variants were classified as likely pathogenic variant and uncertain significance respectively, in combination with the clinical manifestation and pedigree analysis of the examinees.

**Table 1:** Exon detection report

Genes	Location of the HG19	Transcript	Changes of Nucleotide and amino acid	Frequency	ACMG variation	Related disease	Origin
SLC2A10	Chr20:45,354,728	NM_030777	c.1057_1058delCT (p.L353Tfs*8)	< 0.001	Likely pathogenic	Arterial tortuosity syndrome	Mother (Carriers)
SLC2A10	Chr20:45,354,587	NM_030777	c.912 T > G (p.C304W)	< 0.001	Variant of uncertain significance	Arterial tortuosity syndrome	Father (Carriers)

Targeted exome sequencing that included 5,177 genes

The first baby followed up to 19 months, with normal growth and development and had been hospitalized for several times due to severe pneumonia and occasionally cough with dyspnea. Facial characteristic features gradually as aging, such as blepharophimosis or periorbital fullness, downslanted palpebral fissures, micrognathia, large ears, long face and cutis laxa. Echocardiography in child showed the Arch and PA were still tortuous. These extensive tortuous and elongated vessels would be difficult to handle in the operation according to the consultation of pediatric cardiac surgery experts. It has been reported that severe pulmonary stenosis usually requires surgery and/or transcatheter treatment, but aortic arch abnormality does not require intervention in most cases of ATS [4].

**Fig. 3:**
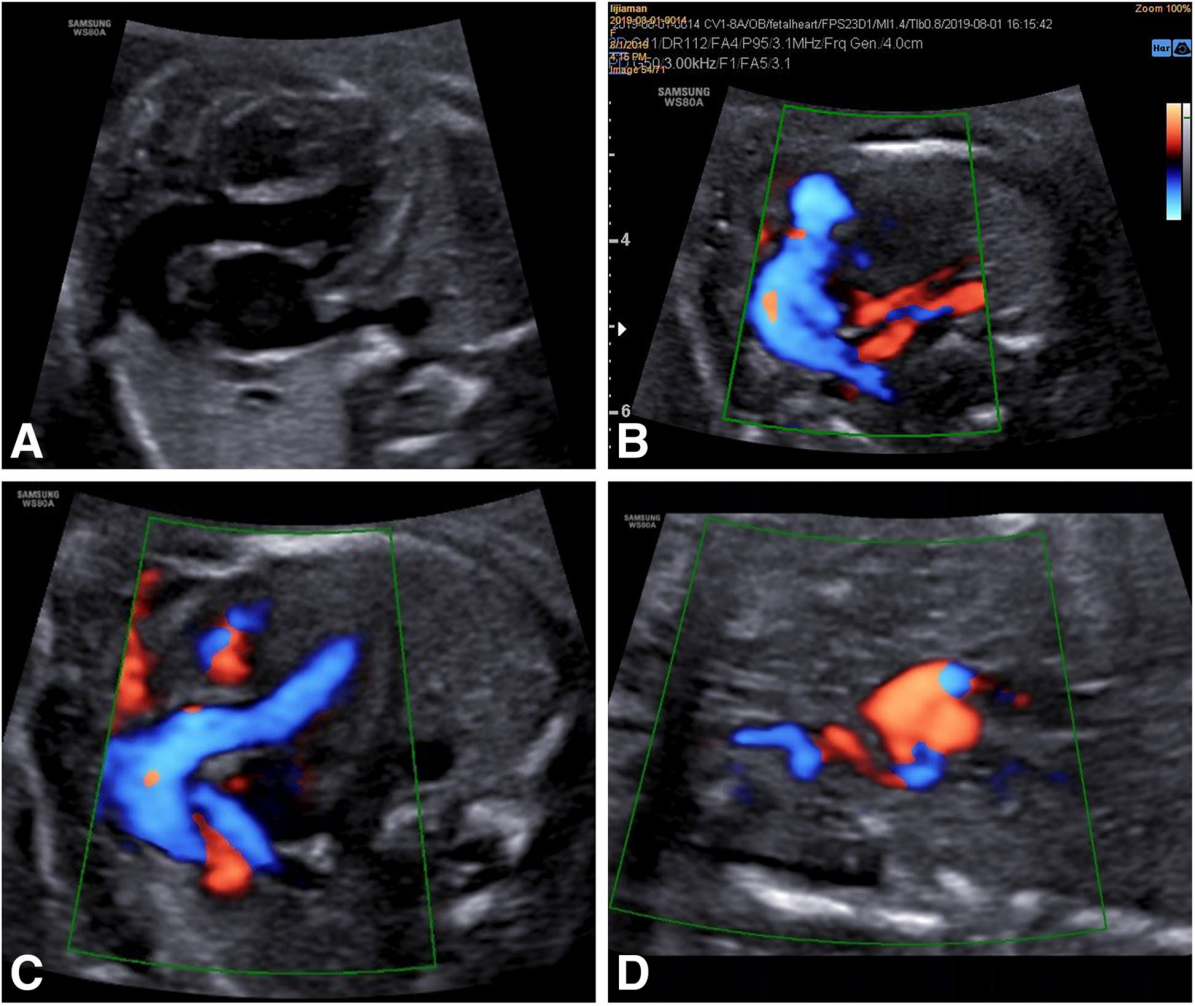
The prenatal ultrasonic image of the second fetus at 22 weeks. **A**, **C** Showing the tortuous and elongated aorta that similar to the images of the first fetus. **B** RPA, LPA and DA are significantly elongated and tortuous. **D** RCA is tortuous originated from IA. The measurement in detail as follows: AAO 0.46 cm, Aortic Arch 0.47 cm, MPA 0.59 cm, RPA 0.18 cm, LPA 0.19 cm, DA 0.19 cm, RCA 0.19 cm. AAO: ascending aorta; DA: ductus arteriosus; MPA: main pulmonary artery; RPA: right pulmonary artery; LPA: left pulmonary artery; RCA: right common carotid artery

Prenatal ultrasound scanning at 22 weeks of gestation of the second fetus showed (Fig. 3) similar echocardiography with tortuous and elongated great arteries. An additional movie files show this in more detail. (See Additional file 4: Video) Parents who continue the pregnancy after prenatal consultation and are fully aware that both parents are carriers have a 25% chance of having the disease in their next child. Doctor suggested a genetic test for the second fetus, and the parents refused to do amniocentesis and also refused to do after birth.

## Discussion

ATS was first described by Ertugul in 1967 [5] and Beuren et al. in 1968 [6]. ATS has no reliable estimate of prevalence. Some authors suggest that it may be more frequent than estimated [7]. However prenatal ultrasound diagnosis of ATS is very rare. Despite few reports of prenatal suspicion of ATS antenatally at 28,29,30 weeks but not have been confirmed after birth [8]. Recently has been reported a case confirmed homozygous for p.S81R pathogenic variant in the SCL2A gene diagnosis of ATS antenatally at 29 weeks and mother was the same variant, the baby appeared well after birth. 11 months of age revealed progressive and severe PA stenosis and taken up for an operation. At 18 months had obviously appearance like our sibling cases [9]. The literature affected the vessels about MPA and their branches and aorta, it’s similar with our cases just elongated and tortuous there was no stenosis noted.

**Fig. 4:**
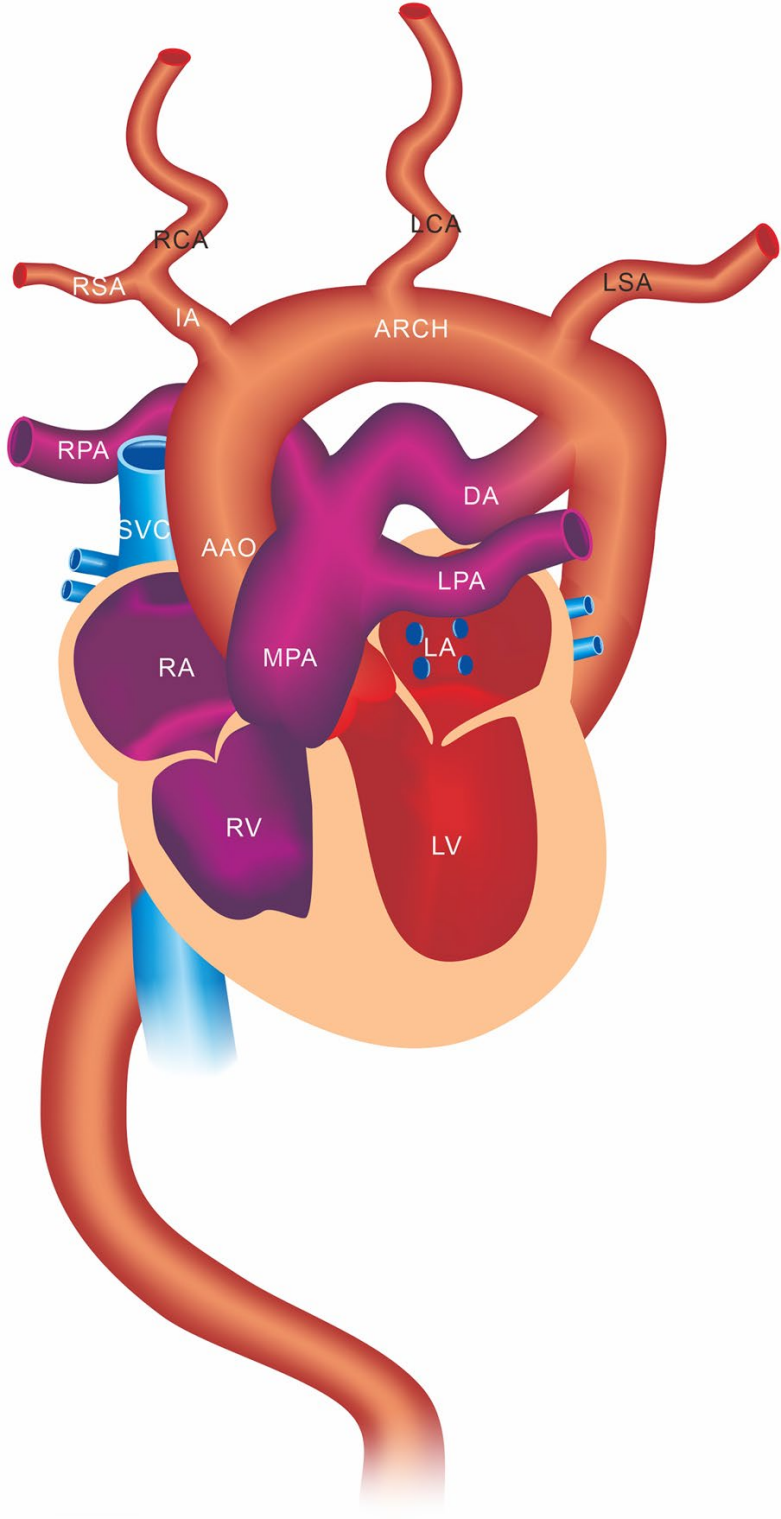
The model diagrams of Arterial Tortuosity Syndrome reveals elongation and tortuosity of the large and medium sized arteries like MPA, LPA, RPA, AAO, Arch, IA, RCA, RSA, LCA, LSA and DAO. MPA: main pulmonary artery; RPA: right pulmonary artery; LPA: left pulmonary artery; AAO: ascending aorta; Arch: aortic arch; IA: innominate artery; RSA: right subclavian artery; RCA: right common carotid artery; LCA: left common carotid artery; LSA: left subclavian artery; DAO: descending aorta

Due to the high suspicion of ATS in prenatal ultrasound and genetic confirmation, we summarized our experience and drew a simulated diagram to facilitate understanding and learning (Fig. 4). Both of our cases presented the same ultrasound appearance on prenatal ultrasound scanning. Fetal echocardiography revealed elongation and tortuosity of the large and medium sized arteries, postpartum CT showed the aorta is tortuous, dilated and elongated with large span, CTA cross-sectional section showed the tortuous and dilated LPA in front of the bifurcated left and right bronchi. The CTA longitudinal section about the first case showed the arch and three branches were abnormally zigzag.

Major clinical findings mainly divided into cardiovascular involvement, craniofacial involvement, skin and connective tissue disorder. In terms of cardiovascular disease, it could happen aneurysms [7, 10–12], pulmonary hypertension [11], large-vein dilation, valvular regurgitation and mitral valve prolapse [13, 14]. The risk is also increased at any age for ischemic vascular events involving cerebrovascular circulation (resulting in non-hemorrhagic stroke) and the abdominal arteries (resulting in infarctions of abdominal organs). Characteristic features including blepharophimosis or periorbital fullness, downslanted palpebral fissures, convex nasal ridge, midface retrusion, micrognathia, large ears, long face, high palate, and dental crowding are often present and become more prominent with aging in the craniofacial involvement. The patients might be present loose skin folds and redundancy as seen in cutis laxa syndromes [7]. Other evidence of a generalized connective tissue disorder includes skeletal manifestations; inguinal and abdominal wall hernias; sliding hiatal or diaphragmatic hernia; hypotonia; myopia; and/or keratoconus [7]. Diaphragmatic hernia and sliding hiatal hernias are reported in up to 50% of affected individuals [15]. Although early reports mentioned 40% mortality before age four years [11], larger series of individuals with a molecularly confirmed diagnosis indicate a milder disease spectrum [7]. The earlier literature may also have been biased toward reporting the more severe end of the phenotypic spectrum. Abdulmohsen et al. published 7 patients at a mean follow up at 17.6 months remaining asymptomatic [16]. The diagnosis of ATS is established in a proband with generalized arterial tortuosity and biallelic (homozygous or compound heterozygous) pathogenic variants in SLC2A10.

ATS should be differentiated from EFEMP2-related cutis laxa, Loeys-Dietz syndrome [17, 18], Ehlers-Danlos syndrome, FBLN5-related cutis laxa, LTBP4-related cutis laxa and Occipital horn syndrome. And also differentiated from pulmonary artery sling antenatally, the cross-sectional view at the level of three vessels which includes both PA branches is useful to detect and color and power doppler may be helpful as well. The difference is that the ATS is only PA tortuous and does not bypass the trachea, but the PA sling is the LPA originated from the RPA, originating initially to the right side of the trachea, and turning sharply to course between the trachea and esophagus prior to joining the left hilum [19]. In conclusion, the cases that both parents were carriers, each sib has a 25% chance of being affected, a 50% chance of being an asymptomatic carrier, and a 25% chance of being unaffected and not a carrier. The first case confirmed ATS and although genetic tests were not performed in the second case, ATS was highly suspected based on sonographic findings, facial features and the two cases from the same parents. Timely diagnostic work-up in patients with ATS is necessary to plan eventual intervention, and hopefully to prevent complications related to the abnormal vasculature [20]. Regular cardiovascular follow up with echocardiography, and MRI-angiography or CT scan with 3D reconstructions from head to pelvis starting at birth or at the time of diagnosis. Under stable conditions, echocardiography could be performed on a yearly basis and MRI angiography or CT scan at least every three years in older children and adults. Avoid the contact sports, competitive sports, isometric exercise, scuba diving, the tobacco and sun tanning [21]. Preconception counseling should include possible pregnancy-associated risks to the mother and medication-associated risks to the fetus. Genetic counseling is the process of providing individuals and families with information on the nature, inheritance, and implications of genetic disorders to help them make informed medical and personal decisions. It is appropriate to offer genetic counseling (including discussion of potential risks to offspring and reproductive options) to young adults who are affected, are carriers, or are at risk of being carriers.

## Conclusions

Extra-cardiac abnormalities are difficult to detect, but the great vessel problems are easy to detect prenatally. In summarize, the key points of prenatal ultrasound diagnosis of ATS are the elongation and tortuosity of the large and medium sized arteries. There are great clinical implication of timely diagnostic work-up in patients with unusual appearance of great vessels of the fetal heart during routine morphology examination.

## Supplementary Information

**Additional file 1**. Fetus echocardiography showing the tortuous process of tracing the course and connecting about the great vessels include AAO, Arch, LPA, RPA, DA.

**Additional file 2**. Spatiotemporal image correlation (STIC) showing the correct vascular connection of the tortuous and elongated great vessels about the first fetus in the 22 weeks.

**Additional file 3**. Spatiotemporal image correlation (STIC) showing another view rotate the STIC images 180 degree to observe the backside about the first fetus in the 22 weeks.

**Additional file 4**. Sonogram showing the the tortuous process of tracing the course and connecting about the great vessels include AAO, Arch, LPA, RPA and DA about the second fetus in the 29 weeks.

## Data Availability

The data supporting the conclusions of this article are included within the manuscript (and its additional files). The authors would like to share video data related to the current study, which could only be used for personal study.

## Abbreviations

ATS: Arterial tortuosity syndrome
WES: Whole exome sequencing
CTA: Contrast-enhanced computed tomography images
AAO: Ascending aorta
RSA: Right subclavian artery
DAO: Descending aorta
DA: Ductus arteriosus
MPA: Main pulmonary artery
Arch: Aortic arch
RPA: Right pulmonary artery
LPA: Left pulmonary artery
IA: Innominate artery
LCA: Left common carotid artery
LSA: Left subclavian artery
T: Trachea
LB: Left bronchus
RB: Right bronchus
